# Putting scaling laws on a physical foundation

**DOI:** 10.7554/eLife.89415

**Published:** 2023-06-14

**Authors:** Yiyang Ye, Jie Lin

**Affiliations:** 1 Center for Quantitative Biology, Peking University Beijing China; 2 Center for Quantitative Biology and the Peking-Tsinghua Center for Life Sciences, Peking University Beijing China

**Keywords:** cell volume, pump leak model, nucleus volume, cell density regulation, scaling laws, mitotic swelling, None

## Abstract

As a cell changes size during the cell cycle, why does its density remain constant?

**Related research article** Rollin R, Joanny J-F, Sens P. 2023. Physical basis of the cell size scaling laws. *eLife* **12**:e82490. doi: 10.7554/eLife.82490.

Different types of cells have different sizes, and the size of a given cell type also changes during the cell cycle. The size of a cell influences its biological functions in many ways: in large cells, for example, the protein concentration in the cytoplasm is reduced, which slows down virtually all biochemical reactions. In small cells, on the other hand, the protein concentration can become so high that proteins cannot diffuse efficiently, which also slows down reactions. Therefore, cell size must be regulated for a given cell type.

Although cell size is influenced by multiple factors – including the amounts of proteins, metabolites, and ions in the cell – some simple and universal scaling laws apply to many eukaryotic cells, including yeast and mammalian cells. In particular, the dry mass of a cell and the volume of its nucleus remain proportional to the volume of the cell as it grows during the cell cycle (Neurohr and Amon, 2020; Cantwell and Nurse, 2019; Webster et al., 2009).

These scaling laws may appear obvious to biologists as they simply suggest that the cell density and the ratio of the nuclear volume and the cell volume remain constant. From a physical perspective, however, these scaling laws are highly nontrivial. For instance, the dry mass of a cell is dominated by large biomolecules such as proteins. In contrast, the wet volume (i.e., the volume of water in the cell) is controlled by the number of small molecules, such as metabolites and ions, in the cell: this is because osmotic balance requires that the chemical potential of the water molecules inside and outside the cell must be equal. Therefore, a constant cell density suggests that protein production and small-molecule synthesis are connected. However, there are also times when the cell density does not remain constant: for example, the cytoplasm is significantly diluted in exceedingly large budding yeast cells (Neurohr et al., 2019). This dilution is thought to trigger senescence, but the mechanism responsible is not clear.

Now, in eLife, Romain Rollin, Jean-François Joanny and Pierre Sens of the Institut Curie report new insights into the scaling laws for cell size (Figure 1; Rollin et al., 2023). Their starting point was a model called the Pump-Leak model (Tosteson and Hoffman, 1960). In this model, certain charged molecules cannot pass through the cell membrane, which creates an imbalance in the osmotic pressure inside and outside the cell. Cells seek to reduce the imbalance caused by these impermeant molecules by using ion pumps to pump ions out of the cell. In the meantime, ions are also able to passively diffuse or “leak” through ion channels in the cell membrane. Throughout these processes, cells also need to maintain electroneutrality.

**Figure 1.**
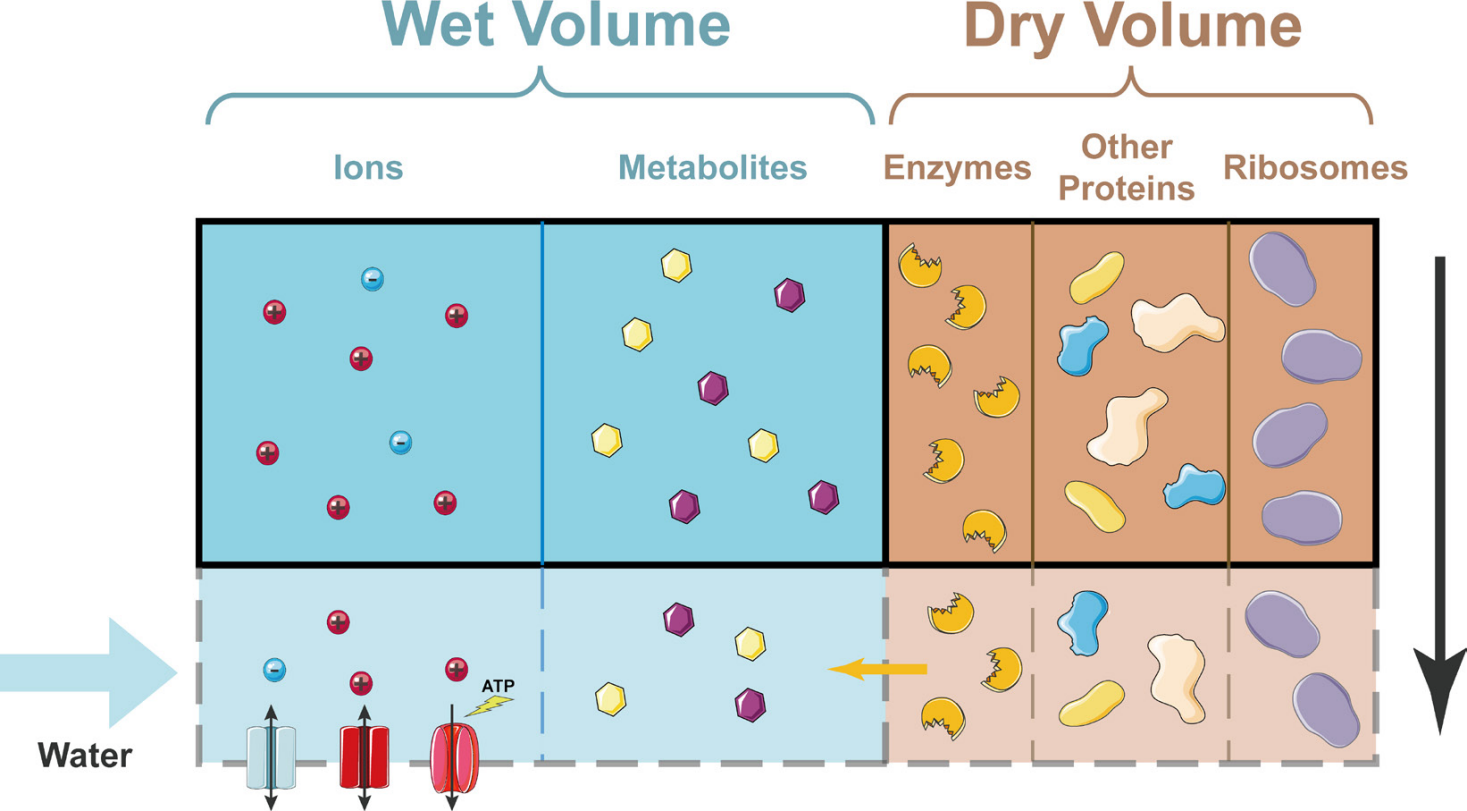
How cell density remains constant as a cell grows. In the simplified model developed by Rollin et al., the dry volume of a cell is dominated by proteins, including ribosomes. Ions can enter and leave the cell through ion channels in the plasma membrane; ions can also be pumped out of the cell by ion pumps (bottom left). Metabolites cannot enter or leave the cell. As a cell grows (shown here schematically by the region inside the solid black line expanding to include the region inside the dashed grey line), the dry volume, which is dominated by proteins, grows exponentially with time. A constant fraction of proteins are enzymes that produce metabolites, so the number of metabolites grows at the same rate. This means that the wet volume, which is approximately proportional to the number of metabolites, also grows at the same rate. Hence, the cell density (which is basically the dry volume divided by the wet volume) remains constant.

Rollin et al. simplified the Pump-Leak model in ways that might seem crude at first sight but actually make the physical basis of cell size much more transparent. In particular, if reasonable assumptions are made about the pumping efficiency, it turns out that the wet volume of the cell is proportional to the number of impermeant molecules, which are primarily metabolites. The researchers also assumed that proteins dominate the dry mass. Furthermore, they included a model of gene expression developed by one of us (JL) and Ariel Amir (Lin and Amir, 2018) and also included protein degradation in their model. This allowed Rollin et al. to demonstrate that the number of metabolites and the total protein content scale with the ribosome number, which grows exponentially with time during the cell cycle. The cell density, therefore, remains constant. However, if the cell size becomes so large that the finite number of DNA molecules in the cell starts to limit the rate of protein production, the amount of protein saturates. However, metabolites are still produced, leading to the dilution of the cytoplasm. Encouragingly, the predictions of this updated Pump-Leak model quantitatively fit data on cell density in budding yeast (Neurohr et al., 2019).

Rollin et al. then explored the rapid swelling that takes places in mammalian cells at the start of mitosis, before being reversed later in the cell cycle (Son et al., 2015; Zlotek-Zlotkiewicz et al., 2015). They propose that counterions released by chromatin are responsible for the swelling, and their estimate for the increase in the cell volume (between ~100µm^3^ and ~150µm^3^) is consistent with the ~10% increase that is typically seen in experiments.

The researchers also demonstrated that metabolites are crucial in setting the ratio of the nuclear volume to the cytoplasmic volume. Based on a nested Pump-Leak model, Rollin et al. went on to show that the ratio of these volumes was equal to the ratio of the protein contents of the nucleus and cytoplasm, so long as the chromatin charge density was low enough. And by demonstrating that cells typically contain a large pool of metabolites, which results in large cell volumes and low chromatin charge densities, they shed light on why the nuclear-cytoplasmic volume ratio remains constant.

By making biologically reasonable assumptions to simplify the classic Pump-Leak model, Rollin, Joanny and Sens have been able to deepen our understanding of various phenomena related to cell size, and to make predictions about the breakdown of the cell size scaling laws that could be tested in experiments.
